# HLA gene expression is altered in whole blood and placenta from women who later developed preeclampsia

**DOI:** 10.1152/physiolgenomics.00106.2016

**Published:** 2017-01-27

**Authors:** Heather Y. Small, Christine Akehurst, Liliya Sharafetdinova, Martin W. McBride, John D. McClure, Scott W. Robinson, David M. Carty, Dilys J. Freeman, Christian Delles

**Affiliations:** ^1^Institute of Cardiovascular and Medical Sciences, University of Glasgow, Glasgow, United Kingdom; and; ^2^Kazan Federal University, Kazan, Russian Federation

**Keywords:** preeclampsia, gene expression, placenta, microarray

## Abstract

Preeclampsia is a multisystem disease that significantly contributes to maternal and fetal morbidity and mortality. In this study, we used a non-biased microarray approach to identify dysregulated genes in maternal whole blood samples which may be associated with the development of preeclampsia. Whole blood samples were obtained at 28 wk of gestation from 5 women who later developed preeclampsia (cases) and 10 matched women with normotensive pregnancies (controls). Placenta samples were obtained from an independent cohort of 19 women with preeclampsia matched with 19 women with normotensive pregnancies. We studied gene expression profiles using Illumina microarray in blood and validated changes in gene expression in whole blood and placenta tissue by qPCR. We found a transcriptional profile differentiating cases from controls; 336 genes were significantly dysregulated in blood from women who developed preeclampsia. Functional annotation of microarray results indicated that most of the genes found to be dysregulated were involved in inflammatory pathways. While general trends were preserved, only *HLA-A* was validated in whole blood samples from cases using qPCR (2.30- ± 0.9-fold change) whereas in placental tissue *HLA-DRB1* expression was found to be significantly increased in samples from women with preeclampsia (5.88- ± 2.24-fold change). We have identified that *HLA-A* is upregulated in the circulation of women who went on to develop preeclampsia. In placenta of women with preeclampsia we identified that *HLA-DRB1 is* upregulated. Our data provide further evidence for involvement of the *HLA* gene family in the pathogenesis of preeclampsia.

of all pregnancies worldwide, 2–8% are affected by the severe form of spontaneous pregnancy-induced hypertension: preeclampsia (29). Preeclampsia is broadly defined by International Society for the study of Hypertension in Pregnancy (ISSHP) criteria as the de novo development of hypertension after gestational *week 20* accompanied by the new onset of one or more of the following: proteinuria, renal insufficiency, liver disease, neurological conditions, hematological disturbances, or fetal growth restriction (35). The disease contributes to maternal and perinatal morbidity and mortality as well as conferring increased long-term risk of chronic illnesses (16, 29). There is a need for greater understanding of the underlying multifactorial development that predates the clinical presentation of preeclampsia. Furthermore, the only cure for preeclampsia is timely delivery of the placenta as well as the baby; therefore, the basis of the disease is thought to be dysfunction of the placenta.

The fundamental changes that occur between a healthy pregnancy and the development of preeclampsia may be associated with changes in gene expression. In particular, examining large-scale gene expression in the circulation provides an accessible source that can be informative of disease states. Previous studies by other groups that have looked at gene expression in whole blood or peripheral blood monocytes (PBMCs) from women with preeclampsia have been analyzed in samples taken at clinical presentation (6, 30, 33) or at delivery (8, 25) and lacked translation into pregnancy-specific tissues (6, 8, 25, 30, 33). In our study, we aimed to analyze gene expression patterns at a time point before clinical presentation of preeclampsia to gain insight into the pathogenic processes that are involved in the development of the condition.

## MATERIALS AND METHODS

### Patients

#### Whole blood microarray cohort.

For the microarray study, whole blood samples from women recruited between 2007 and 2010 as part of the prospective “Proteomics in Preeclampsia” (PIP) study were used. The study protocol has been described elsewhere (5); in brief 4,000 women were recruited across Glasgow at their initial antenatal hospital appointment. Inclusion criteria were as follows: women with singleton pregnancies were recruited at gestational *week 12–16*. Women with a history of chronic hypertension, diabetes, or renal disease were not included in the study. Further samples were obtained from 180 women with two or more risk factors for preeclampsia [nulliparity, age over 35 yr, body mass index (BMI) >30 kg/m^2^, family history of preeclampsia in mother or sister (11)] at gestational *weeks 16* and *28*. All women were followed until delivery, when pregnancy outcomes were obtained from labor ward databases and hospital case records. From this study, whole blood samples taken at 28 wk gestation were obtained in a subset of women who went on to develop preeclampsia (*n* = 5) who were matched for age, BMI, and parity with two normotensive women (controls) per case (total of *n* = 10); these samples were subject to microarray analysis. None of the five women who went on to develop preeclampsia were treated with blood pressure medication at the time of sample collection.

#### Placental tissue cohort.

Placental biopsies were collected at delivery from a second cohort, independently from the PIP study, of 38 women, 19 of whom were diagnosed with preeclampsia according to ISSHP criteria and 19 normotensive subjects matched for age, BMI, and parity. Women were all nonlaboring and underwent caesarean section. None of the women had a medical history of cardiovascular or metabolic disease, and multiparous pregnancies were excluded. Subject characteristics were recorded at the time of tissue collection, and delivery details were recorded from patient notes. Within the subgroup of women who developed preeclampsia: three were treated with labetalol, one was treated with nifedipine, one was treated with betamethasone, and one was treated with betamethasone, labetalol, and nifedipine. Dosage information was not available. The other patients in the cohort did not receive any treatment. Customized birth weight centiles were calculated using the Gestation Network Centile Calculator 5.4 (http://www.gestation.net/birthweight_centiles/centile_online.htm). All biopsies were full thickness; therefore, they incorporated both maternal and fetal tissue. Tissue was flash-frozen in liquid nitrogen and stored at −80°C until analysis.

The PIP Study and the collection of placenta tissue were approved by the West of Scotland Research Ethics Committee and adhere to the principles of the Declaration of Helsinki. All participants gave written informed consent.

### RNA Extraction

RNA extraction from whole blood samples (PAXgene blood RNA tubes, Qiagen) or placental tissues was performed using the miRNeasy mini kit (Qiagen) according to manufacturer’s instructions. Samples were processed according to manufacturer's protocol. Each sample’s total RNA concentration was determined by Nanodrop (Thermo Fisher Scientific) and stored at −80°C until use.

### Illumina Microarray

RNA was amplified and biotin-labeled by in vitro transcription to complementary RNA (cRNA) using the Illumina TotalPrep RNA Amplification Kit (AMIL1791) according to manufacturer’s instructions with 500 ng RNA input. All incubations were performed using an MJ Research PTC Gradient Cycler, and cRNA sample quality was assessed by Agilent 2100 Bioanalyzer before hybridization onto the Illumina Human HT microarray chip (Human HT-12 v4.0 Gene Expression Bead Chip BD-103-0204, Illumina). Samples were loaded to the chips with 750 ng cRNA input and incubated overnight at 58°C. The chips were washed in E1BC buffer, stained with streptavidin-Cy3, and scanned using the Illumina BeadArray Reader. The output data was visualized using Beadstudio software.

### Reverse-Transcriptase Polymerase Chain Reaction

Gene expression reverse-transcriptase polymerase chain reaction (RT-PCR) was performed using the Taqman Reverse Transcription Kit (Applied Biosystems) according to manufacturer’s instructions with 400 ng RNA input. The reaction was run on a Multi Block System Satellite 0.2 Thermo Cooler (Thermo Fisher Scientific) on the following settings: 25°C 10 min, 48°C 30 min, 95°C 5 min.

### Quantitative PCR

Reactions were set up using the following reagents: Taqman Universal Mastermix (Applied Biosystems), dH_2_O, and relevant Taqman probe (Applied Biosystems). Gene expression protocol was run on an ABI PRISM 7900HT PCR system at the following settings: 95°C, 15 min; followed by 40 cycles of 95°C, 15 s; 60°C, 1 min. Threshold cycle (CT) values were analyzed using the 2(−delta delta Ct) method, with dCt indicating normalization to the housekeeper, ubiquitin C (*UBC*).

### Statistical Analysis

The microarray data were preprocessed with GenomeStudio, undergoing background subtraction and quantile normalization. Data were monotonically transformed by taking their logarithm to base 2 after adding a constant to all values. The constant was added to ensure that background subtracted values were all positive and for noise reduction of very low expression probes. Rank products implementation in R was used for significance testing. All *P* values were corrected for multiple testing using false discovery rate (2). Several extreme results had incalculable *P* values, sitting outside of the simulated null distribution, and are described as being less than the lowest calculated value. Quantitative (q)PCR data are represented as means ±  SE and are displayed on the relative quantification scale. Parameters measured in patient cohorts, gene expression changes identified from the microarray and all qRT-PCR data were analyzed using a Student’s *t*-test where significance was defined as *P* < 0.05, and data were analyzed on the delta-CT scale. Pearson correlation was used to estimate linear relationship between gene expression and birthweight or placental weight. Fisher’s transformation was used to test the null hypothesis that population correlation, ρ, equals 0.

## RESULTS

### Whole Blood Samples Taken at 28 wk Gestation in Women Who Went on to Develop Preeclampsia Show a Differential Gene Expression Profile

**Fig. 1.**
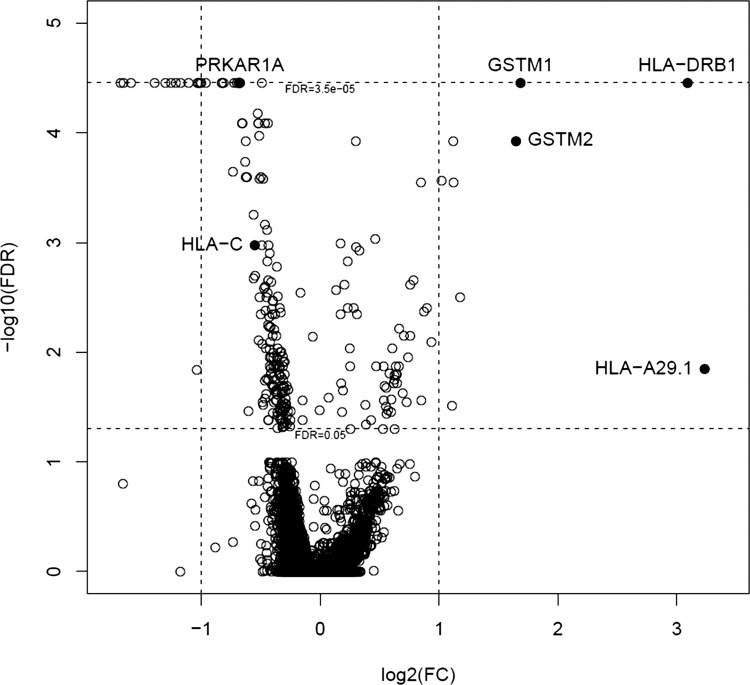
Microarray analysis shows differential gene expression in whole blood samples taken at 28 wk gestation from women who went on to develop preeclampsia. Microarray analysis was conducted in whole blood samples from women who went on to have a normotensive pregnancy (*n* = 10) and women who developed preeclampsia. The microarray data was preprocessed with GenomeStudio, undergoing background subtraction and quantile normalization. Data were monotonically transformed by taking their logarithm to base 2 after adding a constant to all values. The constant was added to ensure that background subtracted values were all positive and for noise reduction of very low expression probes. Rank products implementation in R was used for significance testing. All *P* values were corrected for multiple testing using false discovery rate (FDR); a *P* value of <0.05 was considered to be significant. Black dots (●) accompanied by gene names represent those that were taken forward for Ingenuity Pathway Analysis.

Whole blood samples collected at 28 wk gestation from women who went on to develop preeclampsia (*n* = 5) and women with normotensive pregnancies (*n* = 10) were subject to gene expression analysis using Illumina microarray. Characteristics of this patient cohort are shown in Table 1. Of the 47,000 probes present on the microarray, 336 genes were found to be significantly differentially regulated in women who went on to develop preeclampsia, 163 genes were significantly upregulated, while 173 genes were significantly downregulated (Fig. 1).

**Table 1.** Characteristics of women in the microarray study cohort

	Cases (*n* = 5)	Controls (*n* = 10)	*P* Value
Age, yr	33.2 ± 1.3	33.8 ± 0.5	NS
BMI, kg/m^2^	29.9 ± 0.98	29.2 ± 0.95	NS
Smoker, *n*	0	1 (10%)	NS
Nulliparous	4 (80%)	6 (60%)	NS
Previous preeclampsia	0	0	NS
Family history (mother or sister) of preeclampsia	0	0	NS
SBP 28 wk gestation, mmHg	134 ± 3	113 ± 2	0.044
DBP 28 wk gestation, mmHg	86 ± 3	70 ± 1	0.054
Caesarean section, *n*	1 (20%)	0	NS
Gestation at delivery, wk	38.8 ± 0.6	40.4 ± 0.8	NS
Birth weight, g	3065 ± 120	3358 ± 69	NS

Data are presented as means ± SD. Whole blood samples taken at 28 wk of gestation from 5 women who developed preeclampsia (cases) were matched for 10 women who went on to have normotensive pregnancies (controls). Both systolic and diastolic blood pressure were significantly elevated in cases relative to controls; despite these samples being taken before the onset of labor or diagnosis of preeclampsia. BMI, body mass index; SBP, systolic blood pressure; DBP, diastolic blood pressure; NS, not significant.

**Fig. 2.**
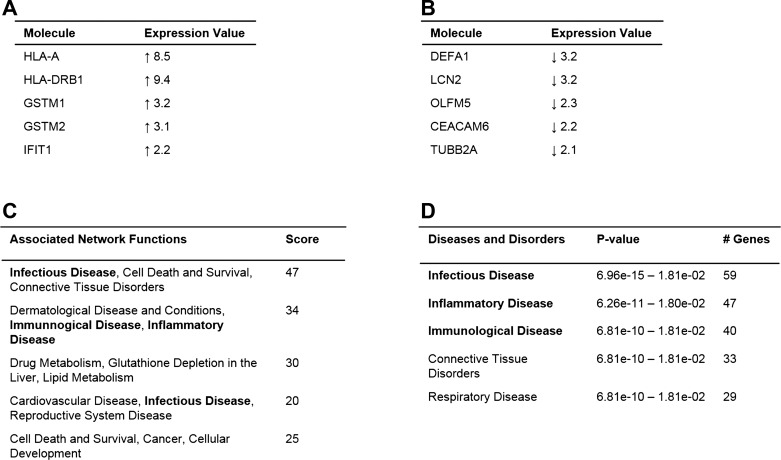
Ingenuity Pathway Analysis (IPA) of genes that were significantly differentially expressed in whole blood samples from women who went on to develop preeclampsia from microarray. *A* and *B*: the genes that had the largest fold change from the microarray; either increased or decreased respectively. Biological pathway search was modified to include direct or indirect relationships where gene involvement had been experimentally validated. Disorders of the immune system featured multiple times in both network analysis (*C*) and in association with disease and disorders (*D*).

Genes that were significantly differentially regulated between the two groups were subject to Ingenuity Pathway Analysis (IPA) to delineate their involvement in biological pathways of interest. Disorders of the immune system were among the main hits from IPA analysis (Fig. 2). We chose five genes, *HLA-A*, *HLA-C*, *HLA-DRB1*, *GSTM2*, and *PRKAR1A*, to take forward for further study on the basis of the highest fold change and/or biological function determined from IPA and a literature search (Table 2).

**Table 2.** Genes of interest identified to be significantly differentially expressed in whole blood samples at 28 wk gestation from women who went on to develop preeclampsia

	HGNC ID	Chromosomal Location	SNPs, *n*	Preeclampsia vs. Control (Log_2_ Fold Change)	Preeclampsia vs. Control (FDR)	Canonical Pathway (Ingenuity Pathway Analysis)	Previous Associations with Pregnancy Outcome	Pregnancy-Specific Changes in Expression
*HLA-DRB1*	4948	6p21.3	1470	9.4	0	graft vs. host disease signaling	miscarriage (12, 31)	not investigated
							birthweight (28)	
							intrahepatic choleostasis (10)	
							inflammatory bowel disease (15)	
*PRKAR1A*	9388	17q24.2	592	−1.6	0	PXR/RXR activation	none	not investigated
*GSTM2*	4634	1p13.3	427	3.1	0.00012	PXR/RXR activation	none	increased by progesterone in preimplantation period in mice (22)
*HLA-C*	4933	6p21.3	1163	−1.4	0.001071	graft vs. host disease signaling	miscarriage (7)	highly expressed in the extravillous trophoblasts (18, 24)
*HLA-A*	4931	6p21.3	1370	8.5	0.014369	graft vs. host disease signaling	miscarriage (7)	not investigated
							birthweight (12, 31)	

Five genes of interest were found to be significantly altered in the circulation of women who went on to develop preeclampsia from microarray analysis. Genes of interest were selected using Ingenuity Pathway Analysis to group genes according to their biological function. Gene identification numbers are taken from the HUGO gene nomenclature committee (www.genenames.org accessed. June 26, 2015). Information regarding chromosomal location and number of single nucleotide polymorphisms (SNPs) was taken from www.genecards.org accessed June 26, 2015. PXR, pregnane x receptor; RXR, retinoid X receptor.

### HLA-A was Significantly Upregulated in Whole Blood from Women Who Went on to Develop Preeclampsia

**Fig. 3.**
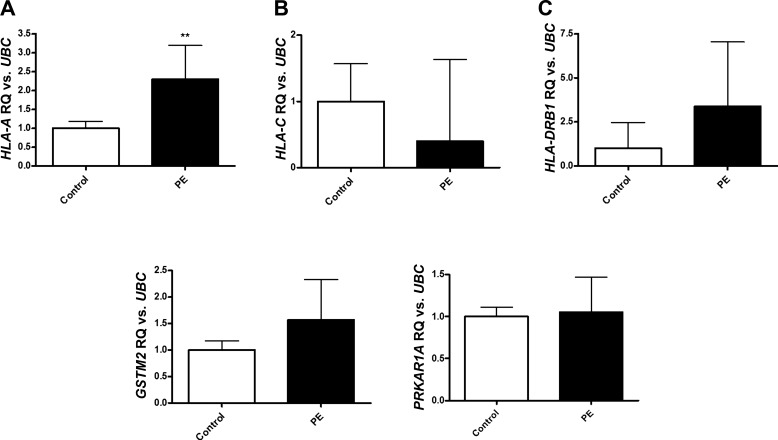
*HLA-A* is significantly upregulated in whole blood samples at 28 wk gestation from women who went on to develop preeclampsia following quantitative (q)PCR validation. Mature mRNA expression of genes of interest were measured by qPCR in whole blood samples from women with preeclampsia (PE) at 28 wk (*n* = 5) and women with normotensive pregnancies (control) (*n* = 10). Data was analyzed by Student’s *t*-test where *P* < 0.05 was considered to be significant (**P < 0.01 vs. control). *HLA-A*, major histocompatibility complex, class I, A; *HLA-C*, major histocompatibility complex, class I, C; *HLA-DRB1*, major histocompatibility complex, class II, DR beta 1; *GSTM2*, glutathione S-transferase mu 2; *PRKAR1A*, protein kinase CAMP-dependent type I regulatory subunit alpha; *UBC*, ubiquitin C.

The genes of interest shown in Table 2 were subject to qPCR validation in the same whole blood samples that were used for the microarray (Fig. 3). While general trends in upregulation and downregulation from the microarray were preserved, only *HLA-A* was found to be statistically significantly upregulated in the qPCR experiment. *HLA-C*, *HLA-DRB1*, *GSTM2*, and *PRKAR1A* were not found to validate at this stage. In agreement with the microarray data, *HLA-A* was upregulated in women who went on to develop preeclampsia but to a lesser extent (log_2_ fold change: 2.30 ± 0.9) than in the microarray experiment.

### HLA-DRB1 is Significantly Upregulated in Placental Tissue from Women with Preeclampsia

**Fig. 4.**
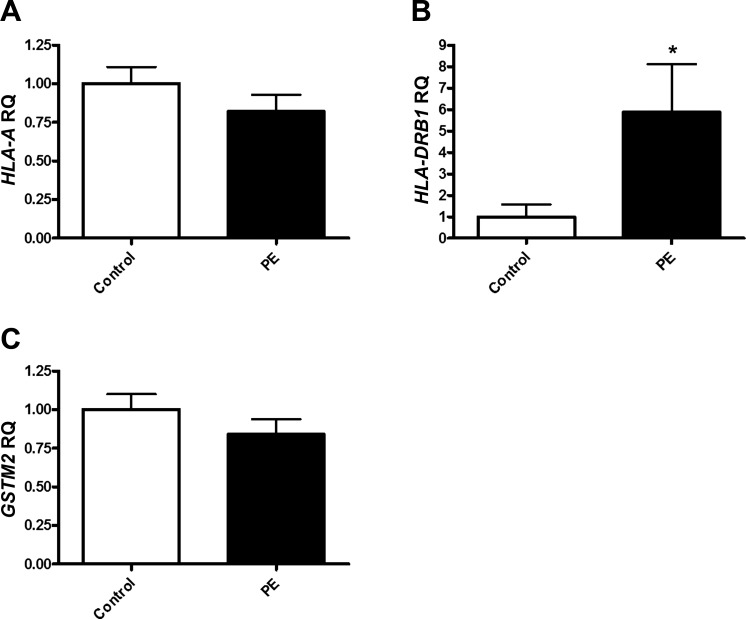
*HLA-DRB1* is significantly upregulated in placental tissue from women with preeclampsia. Mature mRNA expression of genes of interest were measured by qPCR in placental tissue samples from women with preeclampsia (PE) (*n* = 19) and women with normotensive pregnancies (control) (*n* = 19). Data was analyzed by Student’s *t*-test where *P* < 0.05 was considered to be significant (*P < 0.05 vs. control). *HLA-A*, major histocompatibility complex, class I, A; *HLA-DRB1*, major histocompatibility complex, class II, DR beta 1; *GSTM2*, glutathione S-transferase mu 2.

**Fig. 5.**
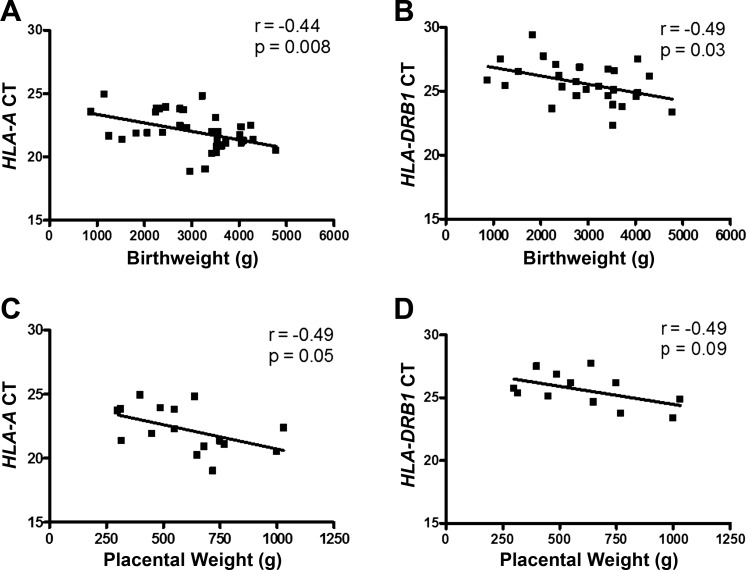
High *HLA-A* and *HLA-DRB 1* expression are significantly correlated with a reduced birthweight but not placental weight. Gene expression values (CT) were subject to Pearson’s correlation and Fisher’s transformation where birthweight (*n* = 28) or placental weight values (*n* = 16) were available. These samples were derived from the placental cohort. *HLA-A* (*A*) CI (−0.67 to −0.12) and *HLA-C* (*B*) CI (−0.69 to −0.06) gene expression were significantly correlated with birthweight but not placental weight (*C* and *D*). A *P* < 0.05 was considered to be significant. *HLA-A*, major histocompatibility complex, class I, A; *HLA-DRB1*, major histocompatibility complex, class II, DR beta 1.

Following the discovery of an altered gene expression profile in the circulation of women who went on to develop preeclampsia, we then sought to investigate a subset of the genes of interest in a tissue with pregnancy-specific relevance. Placental samples were collected at delivery from women with preeclampsia (*n* = 19) and matched women with normotensive pregnancies (*n* = 19) (Table 3). The higher *HLA-A* expression seen in whole blood from cases was not mirrored in the placental tissue (Fig. 4*A*). However, expression of *HLA-DRB1* was significantly increased in placental tissue from women with preeclampsia (fold change: 5.88 ± 2.24) (Fig. 4*B*). We found that placental expression of *HLA-A* (*r^2^ = *0.19, *P =* 0.008) and *HLA-DRB1* (*r^2^ = *0.18, *P =* 0.03) was significantly correlated with birthweight (Fig. 5, *A* and *B*). Where placental weight data were available, *HLA-A* and *HLA-DRB1* were not associated with a reduced placental weight (Fig. 5, *C* and *D*).

**Table 3.** Characteristics of the placental tissue cohort

	Cases (*n* = 19)	Controls (*n* = 19)	*P* Value
Age, yr	29 ± 5.4	30 ± 4.6	NS
BMI, kg/m^2^	29.5 ± 6.8	29.4 ± 6.7	NS
Smoker, *n*	4 (21.1%)	5 (26.3%)	NS
Nulliparous	11	8	NS
Third trimester SBP, mmHg	148 ± 25	123 ± 19	0.001
Third trimester DBP, mmHg	93 ± 16	73 ± 9.4	<0.001
Gestation at delivery, wk	35.9 ± 3.2	39.4 ± 1.5	<0.001
Birth weight, g	2463 ± 840	3573 ± 652	<0.001

Data are presented as means ± SD. Placental samples taken at delivery from 19 women with preeclampsia (cases) were matched for age (29.7 ± 0.7 yr) and BMI (29.4 ± 6 kg/m^2^) to 19 women who had normotensive pregnancies (controls). Women with preeclampsia from this cohort, on average, delivered before full term. BMI, body mass index; SBP, systolic blood pressure; DBP, diastolic blood pressure.

## DISCUSSION

This microarray study identified a number of novel genes that were dysregulated in the circulation of women who went on to develop preeclampsia. Of the 336 genes that were significantly differentially expressed, we selected five on the basis of fold change and biological function. One strength of our study is the translation from whole blood to a relevant tissue involved in the pathology of preeclampsia. Placental dysfunction is regarded as the main cause of preeclampsia symptoms, as delivery of the placenta as well as the baby is the only effective cure for this disease. While significant changes in circulating *HLA-A* were not supported by changes in placental tissue, another gene of interest identified from the microarray, *HLA-DRB1*, was significantly upregulated in placental tissue from women with preeclampsia.

The pregnane-X-receptor/retinoid-X-receptor (PXR/RXR pathway) is involved in the metabolism of xenobiotics through activation of the cytochrome P450 family of enzymes (34). In this study, *GSTM2* and *PRKAR1A* were identified to be differentially regulated from the microarray in whole blood from women with preeclampsia and are an intrinsic part of this pathway. Impaired liver function can be a clinical feature of preeclampsia thus these genes were of potential interest, however, they did not validate at the level of qPCR. The other targets identified from the microarray, *HLA-A*, *HLA-C*, and *HLA-DRB1*, are derived from the human major histocompatibility complex (MHC) known as the human leukocyte antigen (HLA) family in humans. The principle function of the MHC/HLA family is to act in the recognition of “self” through antigen presentation to induce the relevant response from the T cell (27). *HLA-A* and HLA-C encode proteins belonging to class I MHC. Class I MHC are found on the cell surface of all nucleated cells in the body where they function to display cytosolic peptides derived from nonself-proteins. HLA-DRB1 encodes a subunit of an MHC class II receptor. Where class I receptors are ubiquitously expressed; class II receptors are generally restricted to antigen presenting cells.

One gene of those selected from the microarray, *HLA-A*, was validated with qPCR. There are both technical and biological reasons for this disparity between microarray and qPCR data. Technically, despite the use of stringent thresholds, microarray data can produce a high number of false positives (23). A larger patient cohort may also have been able to reduce error in qPCR measurement of gene expression, resulting in more statistically significant changes. Biologically, preeclampsia is a heterogeneous disease encompassing a number of underlying pathologies resulting in distinct clinical outcomes. Early- and late-onset preeclampsia are the extremes of this continuum (36), and differences in gene expression in whole blood samples between the two conditions have been described (6, 8). One limitation with respect to our study is that the clinical parameters of the patient cohort from which the blood samples were collected were more representative of late-onset preeclampsia, whereas the cohort of women who gave placental samples was more representative of severe, early-onset preeclampsia. The heterogeneity of preeclampsia itself and different times of onset across our study cohorts may add a confounding factor with respect to gene expression analysis, increasing the range of values, and therefore error, making it difficult to see clear significant differences.

Pregnancy itself is a major immunological adaptation from implantation to parturition. Mild activation of the immune system throughout the course of pregnancy and a slight increase toward delivery are indicated by a raised white blood cell count and proinflammatory cytokines (26). Recent work in preeclampsia has focused on the role of T cells and adaptive immunity in preeclampsia; both *HLA-A* and *HLA-DRB1* identified from this study are involved in the biology of T cells. While we cannot make any conclusive statements about the underlying mechanism of the greater HLA gene expression in women who went on to develop preeclampsia in this study, further investigation of the interaction between antigen-presenting cells, such as those that express *HLA-DRB*, and T cells should be examined. Overall, our findings are in keeping with a role for T cells and adaptive immunity in the pathology of preeclampsia.

The main effectors of spiral artery remodeling are the fetally derived extravillous trophoblasts; failure of the maternal spiral arteries to remodel is a pathological hallmark of preeclampsia (3). The extravillous trophoblasts are thought to evade the host immune system as they invade the uterine wall partly by expression of a unique combination of MHC class I molecules: *HLA-C*, *HLA-E*, and *HLA-G* (17, 18, 24). Therefore, the expression we detected of *HLA-A* and *HLA-DRB1* in the placenta was most likely to be from maternal not trophoblast tissue. Certain forms of the HLA genes expressed by the extravillous trophoblasts have been previously reported in the literature to have an involvement with preeclampsia. Reduction in *HLA-G*, both maternally through functional single nucleotide polymorphisms in the *HLA-G* gene (14, 19) or reduction in fetally derived trophoblasts (20), has been related to an increased incidence of preeclampsia. Additionally, certain combinations of alleles of the fetal *HLA-C* gene and maternal killer immunoglobulin like receptor (KIR) on NK cells have been shown to have an increased incidence of preeclampsia (13). However, in this study, differential expression of *HLA-C* was not validated with qPCR.

While the HLA genes expressed by the trophoblast have been subject to much study with respect to their involvement in preeclampsia, to date, the two genes that we have validated in this study, *HLA-A* or *HLA-DRB1*, have not previously been associated with the disease. *HLA-A* and *HLA-DRB1* have however been related to a reduced birthweight (4, 28); we also observed a significant correlation between *HLA-A* and *HLA-DRB1* gene expression and reduced birthweight but not placental weight. Both placental weight (9) and birthweight (21) have been found to be significantly decreased in women with preeclampsia. Notably in our cohort, the sample size available for placental weight was considerably smaller than for birthweight, but where data were available for both, the placental weight and birth weight were significantly correlated (data not shown).

Other studies using microarray technology have also identified a distinct profile of gene expression in whole blood (6, 8, 33) or PBMCs (25, 30) from women with preeclampsia. While neither *HLA-A* or *HLA-DRB1* has been previously reported in these studies, genes involved in the immune response have been shown to be differentially expressed in women with preeclampsia (6, 8, 33). In these studies, changes in immune-related gene expression were more apparent in women with severe early-onset preeclampsia. This supports our findings that women with preeclampsia have a significantly different gene expression profile in whole blood specifically with respect to those involved in the function of the immune system. In conclusion, we and others have shown a distinct transcriptional profile in whole blood samples from women with preeclampsia. Currently, preeclampsia is a disease of significant burden on both the mother and fetus without an effective screening method or treatment. Future work should seek to validate as many of these novel targets identified from screens such as ours in larger independent cohorts of patients to assess whether these are indicators of functional pathology on a molecular basis.

## GRANTS

The study was funded by grants from the European Union (EU-MASCARA; project reference 278249) and the Scottish Government’s Chief Scientist Office (reference ETM/196). H. Y. Small was funded by a British Heart Foundation Student Fellowship (FS/12/66/30003). L. Sharafetdinova was funded by a grant from the Russian Ministry of Education and a grant from the Boehringer Ingelheim Funds; the work was carried out according to the Russian Government Program of Competitive Growth of Kazan Federal University.

## DISCLOSURES

No conflicts of interest, financial or otherwise, are declared by the author(s).

## AUTHOR CONTRIBUTIONS

H.Y.S., C.A., and L.S. carried out the experiments.; M.W.M, J.D.M., and S.W.R. analyzed the microarray data. D.M.C. collected clinical samples. D.J.F. and C.D. supervised and designed the study.
